# Characterization and calibration of DECTRIS PILATUS3 X CdTe 2M high-*Z* hybrid pixel detector for high-precision powder diffraction measurements

**DOI:** 10.1107/S1600576724010033

**Published:** 2025-02-01

**Authors:** Gavin B. M. Vaughan, Stefano Checchia, Marco Di Michiel

**Affiliations:** a European Synchrotron Radiation Facility (ESRF) 71 Avenue des Martyrs 38000Grenoble France; SLAC National Accelerator Laboratory, Menlo Park, USA

**Keywords:** powder diffraction, 2D detectors, detector correction, total scattering, hybrid pixel detectors

## Abstract

Silicon-based hybrid photon-counting pixel detectors have become the standard for diffraction experiments of all types at low and moderate X-ray energies. More recently, hybrid pixel detectors with high-*Z* materials have become available, opening up the benefits of this technology for high-energy diffraction experiments. However, detection layers made of high-*Z* materials are less perfect than those made of silicon, so care must be taken to correct the data in order to remove systematic errors in detector response introduced by inhomogeneities in the detection layer, in addition to the variation of the response of the electronics. In this paper we discuss the steps necessary to obtain the best-quality powder diffraction data from these detectors, and demonstrate that these data are significantly superior to those acquired with other high-energy detector technologies.

## Abstract 2

The performance of a high-*Z* photon-counting detector for powder diffraction measurements at high (>50 keV) energies is characterized, and the appropriate corrections are described in order to obtain data of higher quality than has previously been obtained from 2D detectors in these energy ranges.

## 1. Introduction

Two decades ago, area detectors began to supplant point detectors for use in routine powder diffraction measurements. Although the gain in time resolution is considerable when using an area detector rather than a point detector configuration, the losses in data quality can also be considerable. The gains in statistical quality are largely offset by losses due to systematic errors primarily caused by spatial variations in the detector efficiency, which can only partially be corrected. Nevertheless, the temporal advantages mean that the use of area detectors for the entire range of measurements on powders and for total scattering has become widespread.

The principal classes of area detectors used for high-energy studies have been image-plate detectors, CCD/CMOS cameras coupled to a visible light emitting phosphor and ‘flat-panel’ pixel detectors originally developed for medical imaging (Lee *et al.*, 2008 ▸; Chupas *et al.*, 2007 ▸; Daniels & Drakopoulos, 2009 ▸). In recent years, hybrid photon-counting (HPC) pixel detectors based on silicon sensors have supplanted these other technologies for most diffraction applications at low to moderate (<20 keV) energies. This is due to an array of characteristics possessed by pixel detectors which make them attractive for diffraction, as described below. For some time, however, higher-energy applications have not been able to benefit from hybrid pixel technology, as the silicon detectors are essentially transparent to X-rays above about 20 keV (Šišak Jung *et al.*, 2017 ▸). This has changed with the recent development of high-*Z* sensor materials and particularly CdTe. Detectors featuring these materials are now routinely used for scattering experiments at synchrotron sources that necessitate X-ray energies in the range 50–100 keV (Drakopoulos *et al.*, 2015 ▸; Vamvakeros *et al.*, 2016 ▸), although detector performances have generally not yet been fully quantified in detail. It has recently been demonstrated (Krause *et al.*, 2020 ▸) that, with the application of appropriate corrections, these detectors are very interesting for demanding high-energy experiments such as charge-density measurements.

In this article, we will discuss the use of such detectors for high-energy scattering applications for polycrystalline and amorphous materials, focusing on the clear advantages they offer compared with earlier detector technologies, and the appropriate corrections to be applied. All data have been collected at beamline ID15A at the ESRF (Vaughan *et al.*, 2020 ▸), where a DECTRIS PILATUS3 X CdTe 2M detector has been in use for nearly all diffraction applications since 2017. The detector’s characteristics have been extensively exploited for total scattering studies (Kalantzopoulos *et al.*, 2018 ▸; Luo *et al.*, 2018 ▸; Amidani *et al.*, 2020 ▸; Estevenon *et al.*, 2023 ▸; Yildirim *et al.*, 2023 ▸; Pokratath *et al.*, 2023 ▸; Cerantola *et al.*, 2023 ▸; Grünewald *et al.*, 2022 ▸; Poonkottil *et al.*, 2022 ▸), X-ray diffraction computed tomography (XRDCT) (Vamvakeros *et al.*, 2018 ▸; Wragg *et al.*, 2021 ▸; Jensen *et al.*, 2022 ▸; Sottmann *et al.*, 2022 ▸; Heenan *et al.*, 2023 ▸), time-resolved diffraction studies (Liu *et al.*, 2018 ▸; Schultheiß *et al.*, 2018 ▸; Clarke *et al.*, 2021 ▸; Slabki *et al.*, 2021 ▸; Li *et al.*, 2023 ▸) and others.

## 2. Comparison of different 2D detector technologies

2D detectors, in the form of photographic film, have been used since the beginning of X-ray diffraction. The earliest modern 2D detectors, image plates, were also offline, as they had to be scanned and regenerated after each exposure. In the 1990s, the first online 2D detectors became available, typically CCD cameras coupled to a fluorescent screen via focusing optics, image intensifiers, lens optics or fiber-optic bundles. All of these coupling schemes introduced significant difficulties from the point of view of X-ray diffraction, due to either low quantum efficiency or significant distortion effects which complicated the acquisition of data with adequate spatial resolution.

More recently, flat-panel charge-integrating detectors, originally designed for medical imaging, have become more prevalent in high-energy X-ray scattering applications. These detectors have very high efficiency, but suffer from high and variable dark currents which leads to an effective reduction of the dynamic range.

HPC pixel detectors (Förster *et al.*, 2019 ▸; Brönnimann & Trüb, 2020 ▸; Andrä, 2021 ▸) have the same high sensitivity as flat-panel detectors and in addition overcome almost all of the problems associated with the above detector technologies. Pixel detectors can have high dynamic range and rapid parallel readout and, furthermore, can be used in photon-counting mode, making the dark current irrelevant and allowing the simple calculation of accurate counting statistics, a perennial problem with integrating area detectors. This also means that repeated acquisitions can be summed without any loss of data quality, allowing further extension of dynamic range. The detector is routinely used in the energy range 50–100 keV, and data have been collected up to 125 keV (Gerber *et al.*, 2020 ▸). A comparison of the different detector technologies is given in Table 1 ▸.

In this article we will discuss the DECTRIS PILATUS3 X CdTe 2M detector (Šišak Jung *et al.*, 2017 ▸; Donath, 2019 ▸). This detector is based on the PILATUS3 platform (Loeliger *et al.*, 2012 ▸) which has a proven history of electronic stability. The electronic scheme used by pixel detectors allows both single-photon counting and energy discrimination, as incident X-rays are detected via the charge generated in the sensor layer (Bergamaschi *et al.*, 2015 ▸). The energy threshold is thus set at half of the incident energy to ensure that incoming photons are counted exactly once. An important side effect of energy thresholding is that both electronic noise and signals from photons with less than half of the energy of the primary beam are eliminated. Practically, this means that, for high-energy operation, the fluorescence from the first several rows of the periodic table is completely eliminated. For example, at an incident energy of >70 keV, all *L* and *M* lines are suppressed, as well as the *K* lines from the first- and second-row transition metals. As the fluorescence signal can be several times stronger than the diffracted signal at high momentum transfer (*q*), this is a considerable advantage, particularly for total scattering applications.

**Figure 1:**
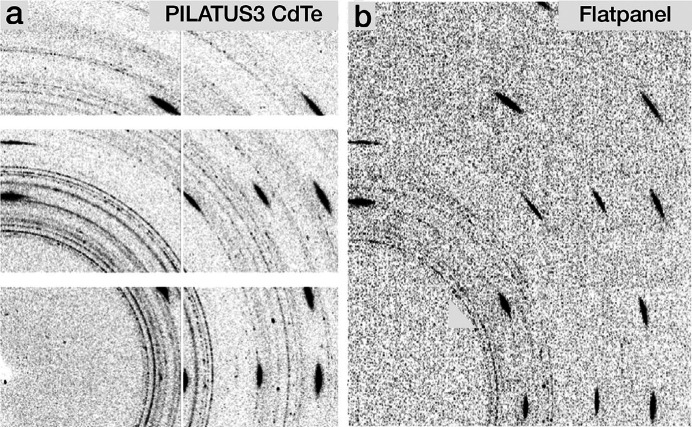
Comparison of data taken with (*a*) the PILATUS 2M and (*b*) a PerkinElmer 1621 from a superconducting filament containing Nb_3_Sn powder in a tungsten tube (diameter 50 µm).

Fig. 1 ▸ shows static powder diffraction patterns recorded at ESRF ID15A at 46.3 keV, comparing data taken with the PILATUS3 X CdTe 300K and a PerkinElmer XRD 1621 CN3 ES flat-panel detector at the same solid angle per pixel and the same exposure time. The sample was a Nb_3_Sn powder contained in a W capillary. The weak signal from the Nb_3_Sn was impossible to measure with the flat-panel detector but is clearly visible in the PILATUS data. Table 2 ▸ gives the values of parameters associated with the two detectors.

## 3. Hybrid pixel detector corrections

In order to fully profit from the excellent characteristics offered by these latest-generation detectors, it is necessary to carefully characterize the detector response to correct for spatial and intensity aberrations (Ruat & Ponchut, 2012 ▸; Grimm, 2020 ▸). In this section we will describe the corrections necessary to recover full data quality.

### 3.1. Masking pixels

#### 3.1.1. Gaps

Unlike silicon, CdTe modules can be made only up to a size of 3 inches (Brönnimann & Trüb, 2020 ▸) and so 3 × 8 modules must be combined to make up the active area of the PILATUS 2M. Even then, modules cannot be arranged contiguously as a region about 3 mm wide is reserved for leakage current collectors, the periphery of the application-specific integrated circuit and wire bonding (Andrä, 2021 ▸). Due to the modular nature of the detector, gaps exist between the different modules, and these must be masked during data treatment, *i.e.* their values excluded from the azimuthal regrouping.

**Figure 2:**
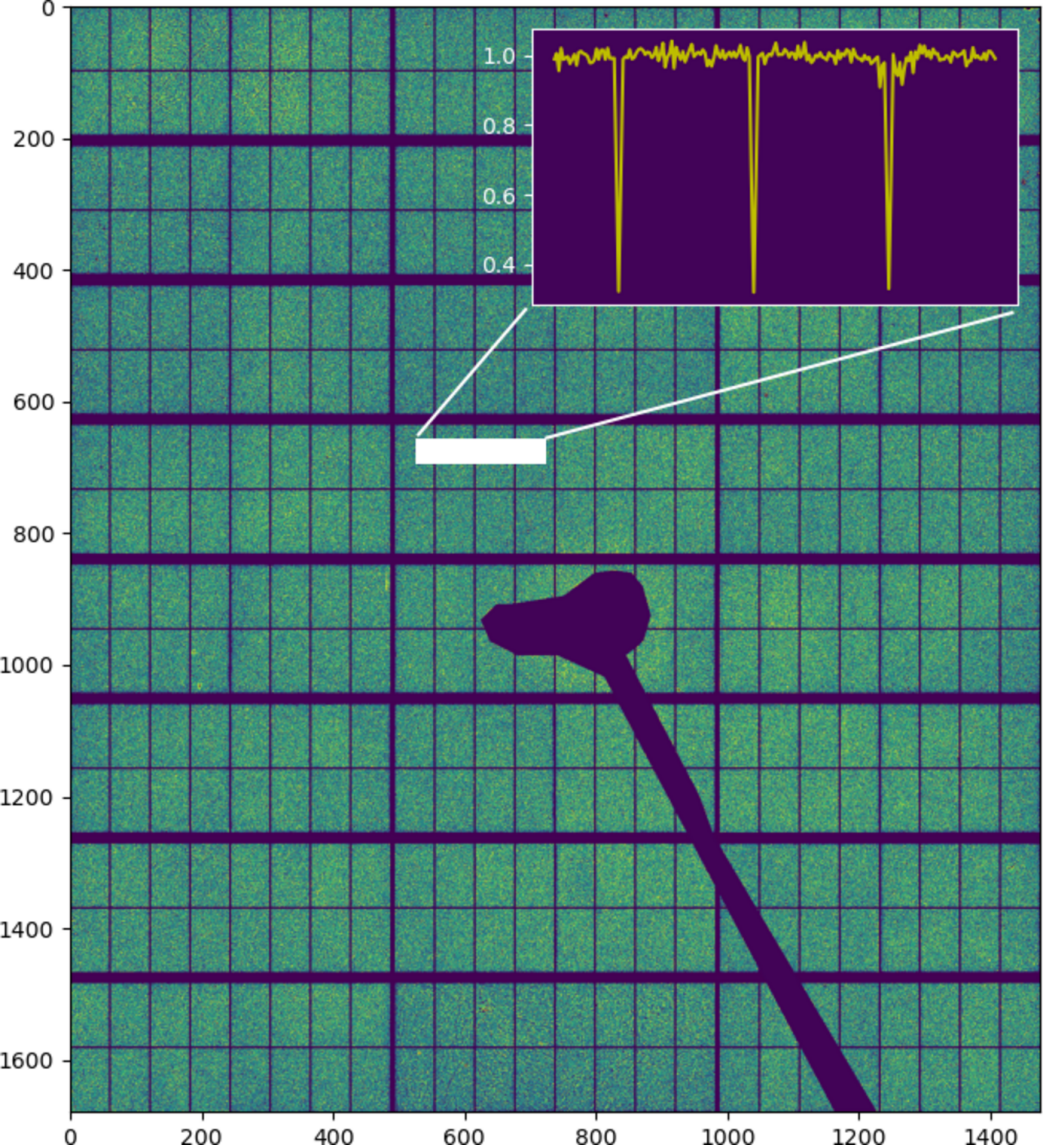
Image of the variance over the mean counts per pixel, measured pixel by pixel for 256 images of silicone oil. The inset shows the altered statistics of the rebinned pixels.

Each CdTe module contains eight sectors; the lines between them are physically larger pixels which have been rebinned in the software to give pixels of apparently the same size as the rest. In doing so, the counts are redistributed equally amongst the new pixels. This has the result of giving a variance in those pixels which is lower than it would be from Poisson statistics; they are not in fact independently measured. The rebinned pixels thus display a variance ranging from 2× (at the edge) to 9× lower at the junctions between lines (Fig. 2 ▸). Additionally, and more seriously, both a non-uniform resolution and diffraction peak shifts result from these pixels, so in practice they are removed from the data.

#### 3.1.2. Defective pixels

**Figure 3:**
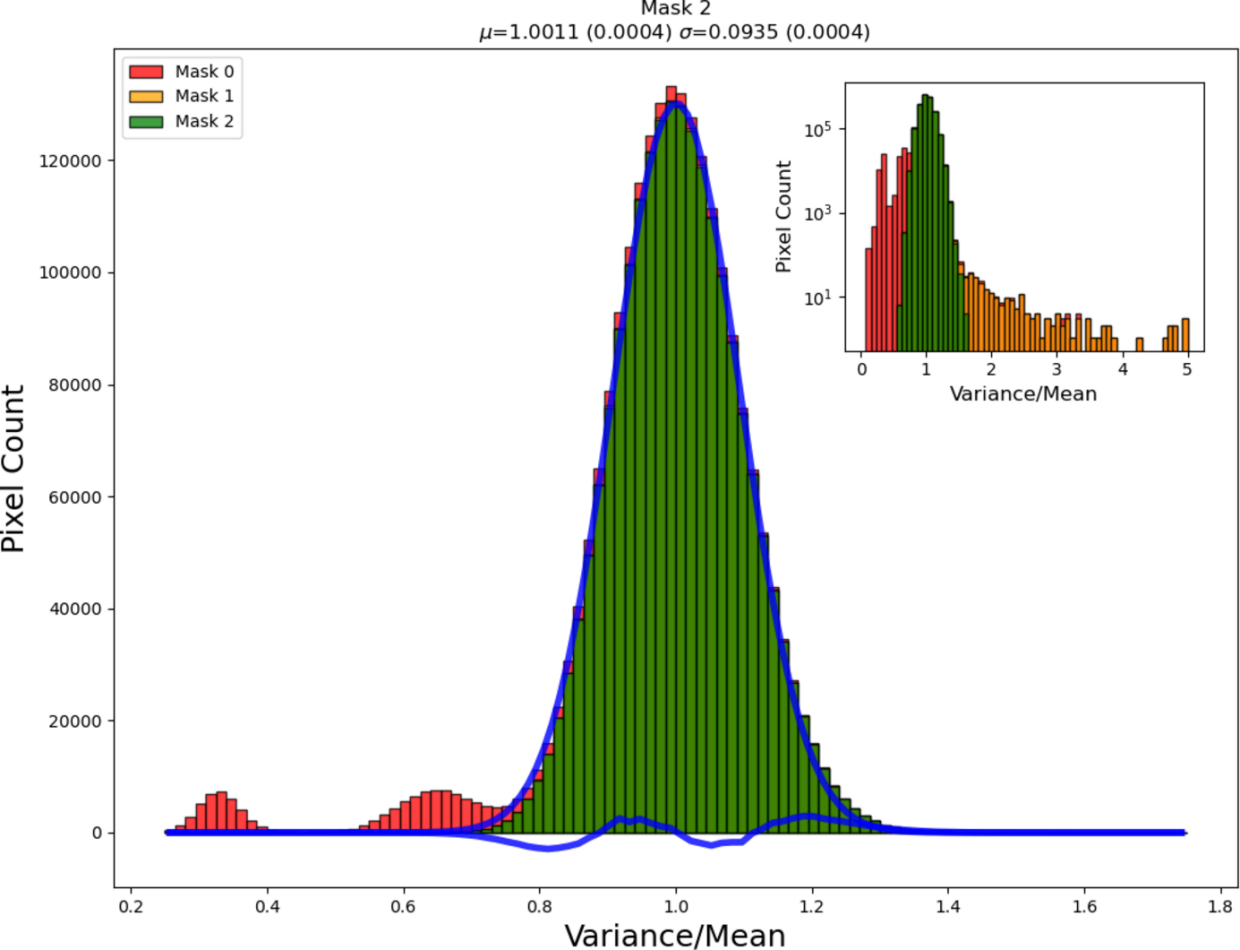
Effect on the counting statistics over the detector by appropriate masking of non-Poissonian and multiple pixels. The distribution of the variance over the mean counts per pixel, measured pixel by pixel for 256 images of silicone oil. Masks 0, 1 and 2 correspond to masking only the pixels flagged by DECTRIS, masking also the rebinned pixels between sub-modules and detector edges, and masking also pixels with high σ/sqrt(mean) (typically damaged pixels) as well as dilating by 1 pixel the regions around each masked pixel, respectively. The percentages of pixels masked in each case were 8.6%, 15.4% and 15.8%, respectively. The blue lines are a Gaussian fit to the final (mask 2) data and the difference curve. Inset: the same data on a semi-log scale.

Due to the difficulty in growing CdTe with the same perfection as Si wafers, several pixels on the detector are defective, exhibiting high noise or non-Poissonian counting, and must be eliminated prior to analysis. The detector was characterized after fabrication and delivered with a mask file in which these pixels are flagged; they are simply masked during data reduction. Further pixels can become damaged over time (from *e.g.* overexposure) and other pixels are observed to have unusually high noise (for example, pixels near the gaps and the detector edges). It is therefore necessary to periodically check the detector for bad pixels; this can be done while carrying out flood field corrections (see below). The effect of removing the defective and the multiple pixels on the global statistics can be seen in Fig. 3 ▸.

### 3.2. Transparency

#### 3.2.1. Intensity correction for incident angle

**Figure 4:**
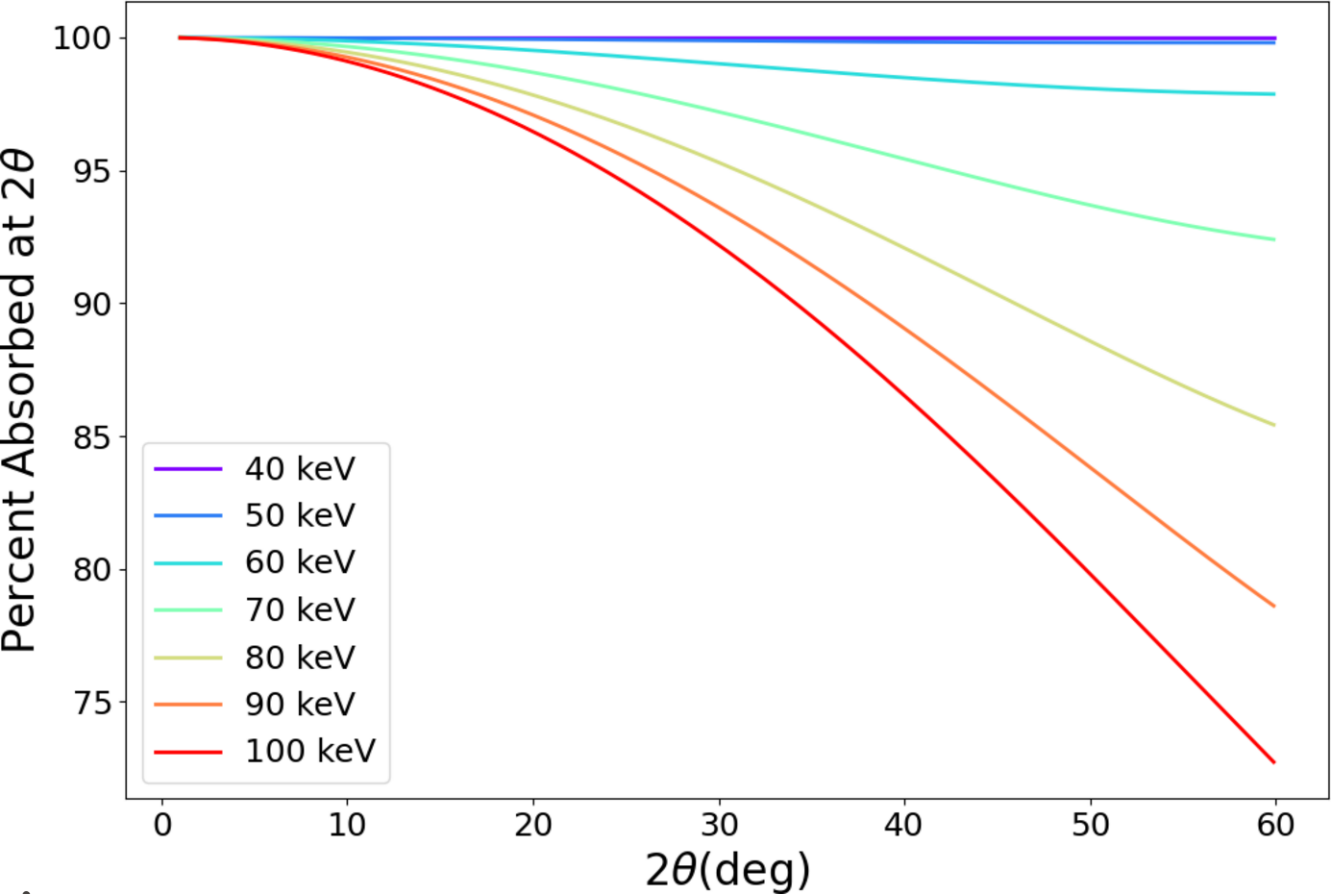
Incident-angle-dependent absorption correction in 2θ for the angular range typically used in diffraction experiments.

The scattering intensity measured by a thick detector must be corrected for the energy- and angle-dependent path length the X-rays travel in the detection layer. If the detector is perpendicular to the beam, this correction is simply scattering angle dependent and can be approximated by the equation
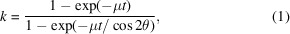
where μ is the X-ray mass attenuation coefficient of the detector medium (CdTe in this case), *t* is the thickness of the detection layer and 2θ is the scattering angle. In this case, the correction has only 2θ dependence and can be applied at any time during the data reduction process. If the detector is not perpendicular, the correction takes a more complex form and must be applied to the 2D data. Fig. 4 ▸ shows the magnitude of the incident angle correction for a perpendicular detector versus 2θ for different energies.

#### 3.2.2. Peak shape and position

The peak shape for powder diffraction data taken with a 2D detector is given by a combination of intrinsic sample broadening, point spread function (in the case of hybrid pixel detectors, essentially the pixel size), sample size, beam size, incident beam bandwidth and divergence, and thickness of the detection layer (Chernyshov *et al.*, 2021 ▸).

**Figure 5:**
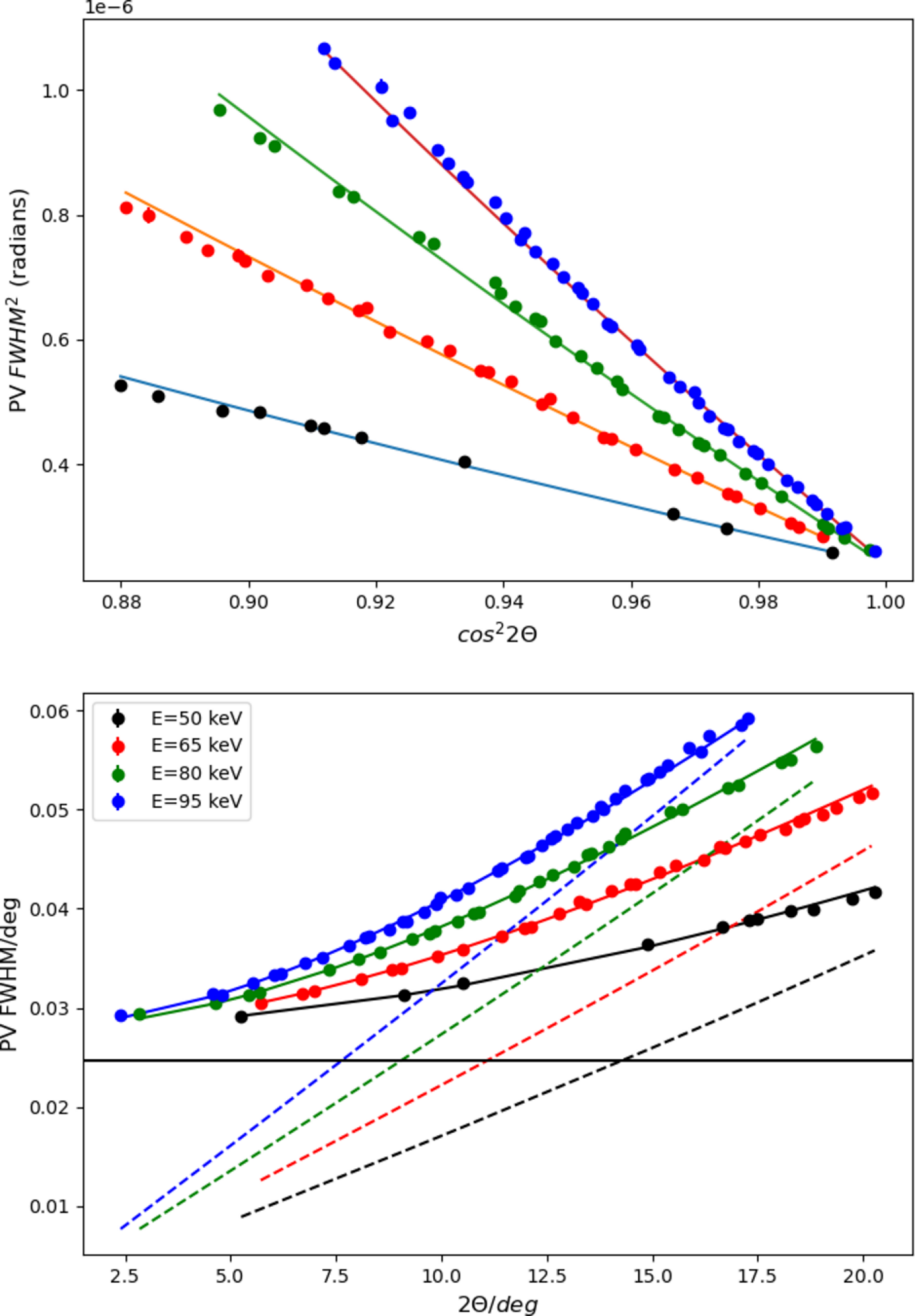
CeO_2_ pseudo-Voigt peak width versus diffraction angle for four different energies, shown as a function of cos^2^ 2θ and 2θ. The circles are data, and the solid lines are fits to a quadratic in cos^2^ 2θ, as described by Chernyshov *et al.* (2021 ▸). The dotted lines in the lower panel represent the calculated contribution from the bandpass of the monochromator, and the nearly horizontal line that of the pixel size, to the resolution function. These two terms are dominant over the effect of detector transparency, too small to be fitted accurately.

The path length of the photon in the CdTe causes a blurring of the angular resolution as a photon incident on a particular pixel on the detector face may traverse several pixels before being absorbed; the effect increases at higher energy and higher angle to a maximum path length of 6 pixels at 100 keV and 45° 2θ, the most extreme conditions in which the detector is used. Measurements of standard materials up to high angles (Fig. 5 ▸ and Fig. S1 in the supporting information) show that this effect is negligible in the current case, where the peak widths are dominated by bandpass, although it is measurable with lower bandpass (Chernyshov *et al.*, 2021 ▸). Furthermore, diffraction patterns of moderately crystalline material are slowly varying at high angle and energy, such that the parasitic contribution from a neighboring pixel is essentially equivalent to that of the pixel in question, rendering this effect effectively negligible.

A secondary effect of the transparency of the detection layer is an angle-dependent increase in peak width and peak asymmetry. To first order, the peak shift is proportional to diffraction angle and thus appears as a modified sample–detector distance, which is corrected by the calibration of the detector geometry. A second-order effect causes peak shifts to higher angle. Fig. S2 shows that this effect is very small (<10^−4^Δ*q*/*q*) even in the most unfavorable conditions used.

### 3.3. Spatial distortion/misalignment

As a pixel detector is a direct detection device, there is no distortion due to detector optics coupling the detector to the detection medium (*i.e.* lenses, image intensifiers or fiber-optic bundles). However, the individual modules which make up the detector have a certain degree of misorientation; this can be detected in high-resolution diffraction patterns, especially when comparing patterns across different azimuthal ranges. The primary module misorientation in the PILATUS detector consists of a rotation around the normal to the module surface (tilt) and a vertical and horizontal displacement with respect to the ideal orientation/position. Tilts out of the detector plane have an immeasurably small effect on the resolution. In order to characterize and correct the module misorientation, a calibration mask has been manufactured and used to measure the misorientation of the modules. This corresponds to the classical method as proposed by Hammersley *et al.* (1994 ▸), Hammersley (2016 ▸) and He (2018 ▸). Alternative methods also exist, for example via the refinement of diffraction patterns (Wright *et al.*, 2022 ▸).

The calibration mask consists of a 500 µm-thick Cu foil on which a regular 2D Cartesian grid of holes has been produced. The hole diameter is 500 µm and their spacing is 5 mm, with a maximum deviation from the ideal position of 8 µm.

**Figure 6:**
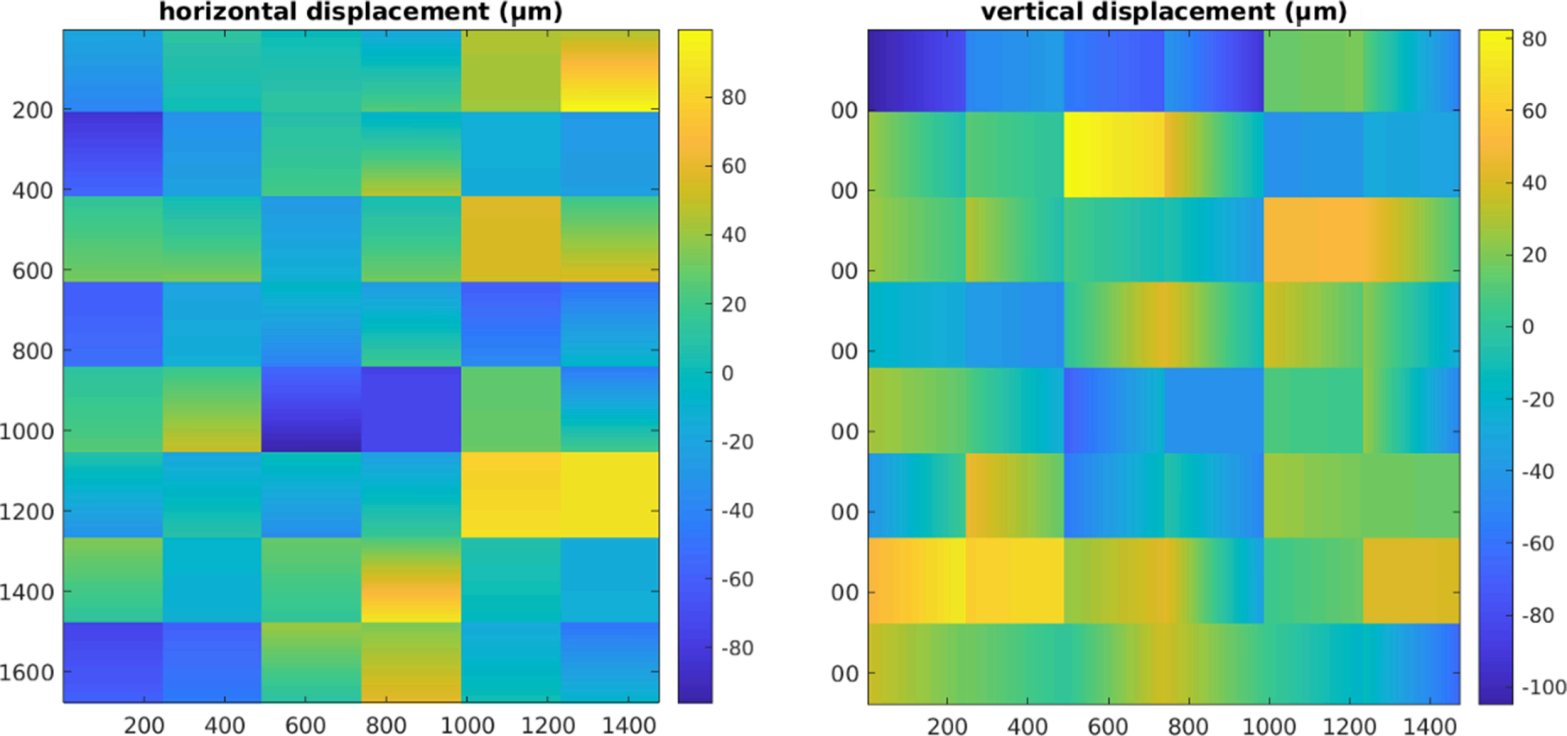
Horizontal and vertical pixel displacement maps measured with a calibration mask. Displacements are given in micrometres.

To measure module misorientation through the calibration mask, a diffraction pattern produced by an amorphous sample was recorded at 50 keV with the mask placed in front of the detector. A second diffraction pattern was recorded without sample and used for intensity normalization. The position of the holes was determined using their center of mass. Comparing the hole positions measured by the detector with the known position on the mask, one can calculate the vertical and horizontal displacement and tilt of each individual module. From those three parameters, by simple trigonometric calculation, the vertical and horizontal displacement of each pixel was obtained. The result is shown in Fig. 6 ▸, in which both the translation (mean value) and rotation (range over a module) are described.

### 3.4. Detector response correction

#### 3.4.1. Static correction

The detector response (‘flood/flat field correction’) is measured by the manufacturer in a limited range of incident beam conditions and may be automatically applied during data acquisition. However, the detector response is observed to vary with time. Furthermore, the manufacturer-supplied corrections are inter/extrapolated between two X-ray energies. Careful measurements indicate that the supplied corrections are not adequate over time, and that indeed the imprecision of this correction is ultimately the limiting step in data quality, eclipsing the effect of counting statistics after even short acquisition times.

Additional flood field corrections have thus been carefully determined over a range of energies and should be applied during data treatment. Ideally, the flood field would be measured by illuminating the detector with a homogeneous source. This is however not generally possible in the practical sense, so the corrections have been generated by the following procedure based on displacing the detector in order to remove the effect of the inhomogeneity of the incident signal. Corrections using a similar rationale have been discussed elsewhere (Kato *et al.*, 2019 ▸; Weng *et al.*, 2023 ▸).

**Figure 7:**
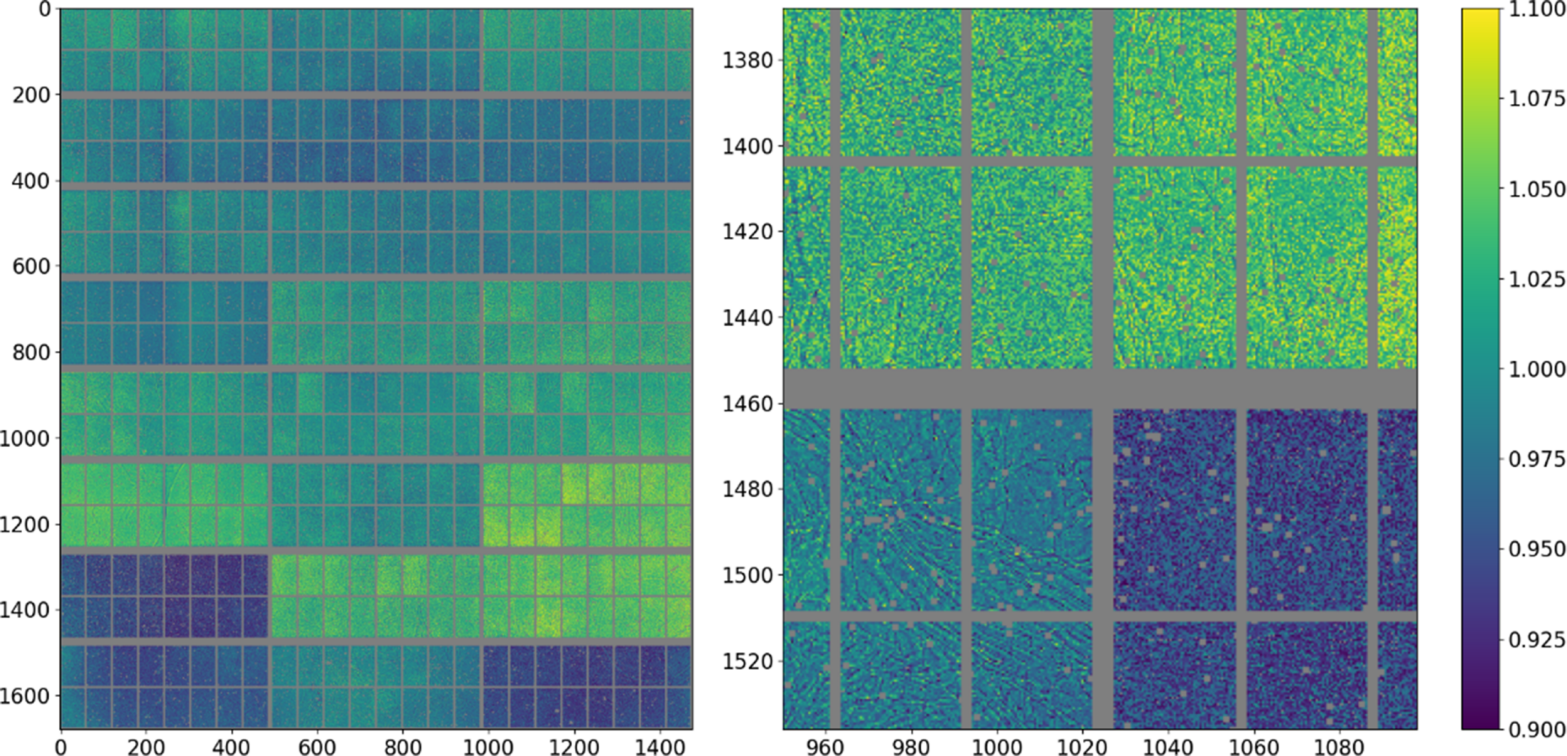
Flood correction at 65 keV. The distribution of values in the correction reflects to first order the variation of response between the different modules, although there are also significant variations within a given module (somewhat correlated with the submodules) and much higher frequency and significant variations (more than 10%) associated with microstructure in the CdTe.

An amorphous scatterer was placed in the beam, and the detector was positioned at high angle and distance from the sample, such that the measured pattern is only slowly varying. Diffraction patterns were collected at 10 keV intervals between 50 and 100 keV at a number of positions (32 patterns at each of 64 positions), where each position corresponds to a displacement of an integer number of pixels. The mean of the 32 patterns is calculated, the data are corrected for incident angle and shifted by the appropriate number of pixels such that the patterns overlap, and each column of the 64 patterns is then simultaneously fitted against a polynomial (a fourth-order polynomial is sufficient). The ratio of the value of each pixel and the model value is the pixel response. By this method, each pixel is measured up to 64 times (less for those close to the edges of the detector) and the values derived are averaged together. Subsequently, outliers in particular patterns are eliminated and the fits are repeated until the values converge to give the flood field correction. By this method, a very high precision can be achieved as not only is the signal measured to a high statistical value but the response of each column of 1675 pixels is fitted with only five parameters, giving a much better result than using counting statistics alone. Furthermore, the fit is model free beyond assuming a smoothly continuous signal. An example of such a correction is shown in Fig. 7 ▸. The other obtained response corrections are shown in Fig. S3. The detector response at other energies is obtained by a cubic interpolation between the measured values.

**Figure 8:**
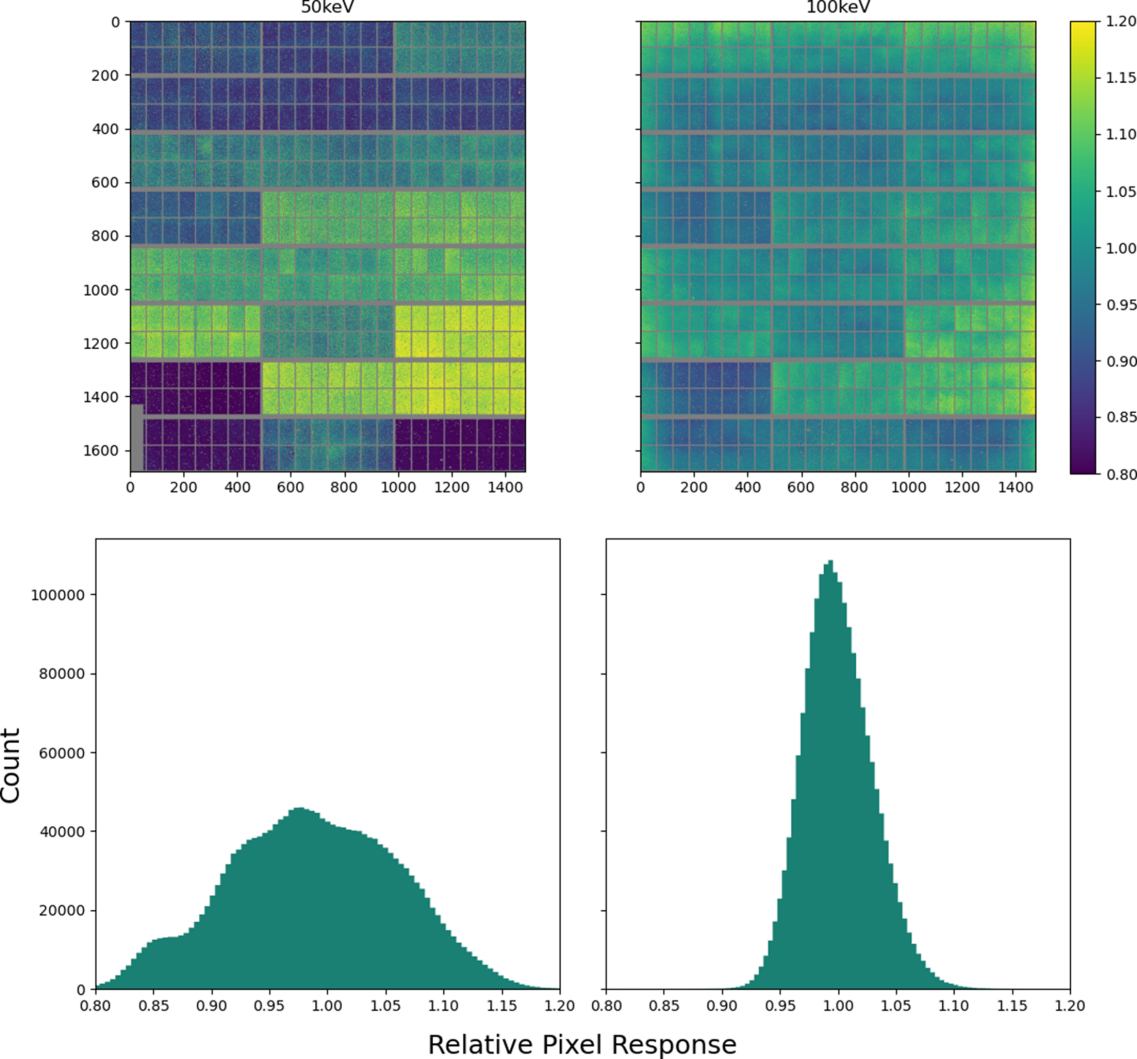
The flood field corrections at 50 and 100 keV. Top: the correction factor for each pixel. Bottom: the distribution of correction factors over the entire image.

**Figure 9:**
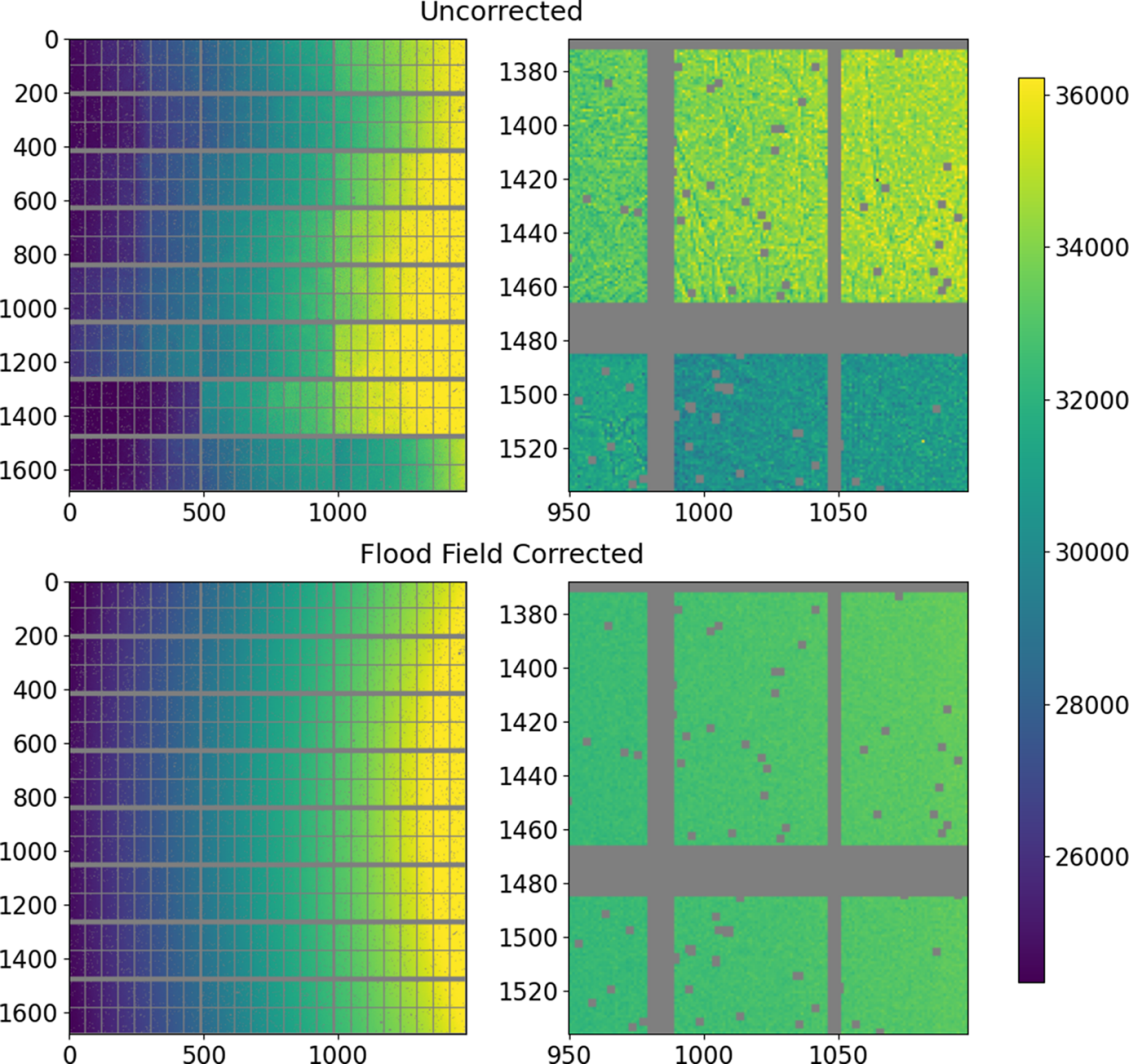
Effect of applying the flood field to high-angle scattering data taken at 65 keV. Above are the data with only the DECTRIS flood field correction; below are the data after application of the supplementary flood field correction collected as described here.

The distribution of responses along the energy range (Fig. 8 ▸ and Fig. S4) sharpens at higher energy. Fig. S5 shows the variation of the responses of several random pixels as a function of energy. The responses are smoothly varying (and generally tend towards the global mean value at higher energy), indicating that interpolation between the different flood fields at intermediate energies gives a valid correction. The ultimate effect of applying the flood correction is shown in Fig. 9 ▸.

#### 3.4.2. Time dependence of detector response

The response of each pixel in the PILATUS varies as a function of time and dose, with the microstructure of the CdTe layer above it strongly affecting the time evolution. Fig. S6 shows the signal from the detector during prolonged exposure to scattering from a sample of water. In the initial image, the microstructure of the CdTe is barely visible, whereas it becomes evident rapidly with exposure.

**Figure 10:**
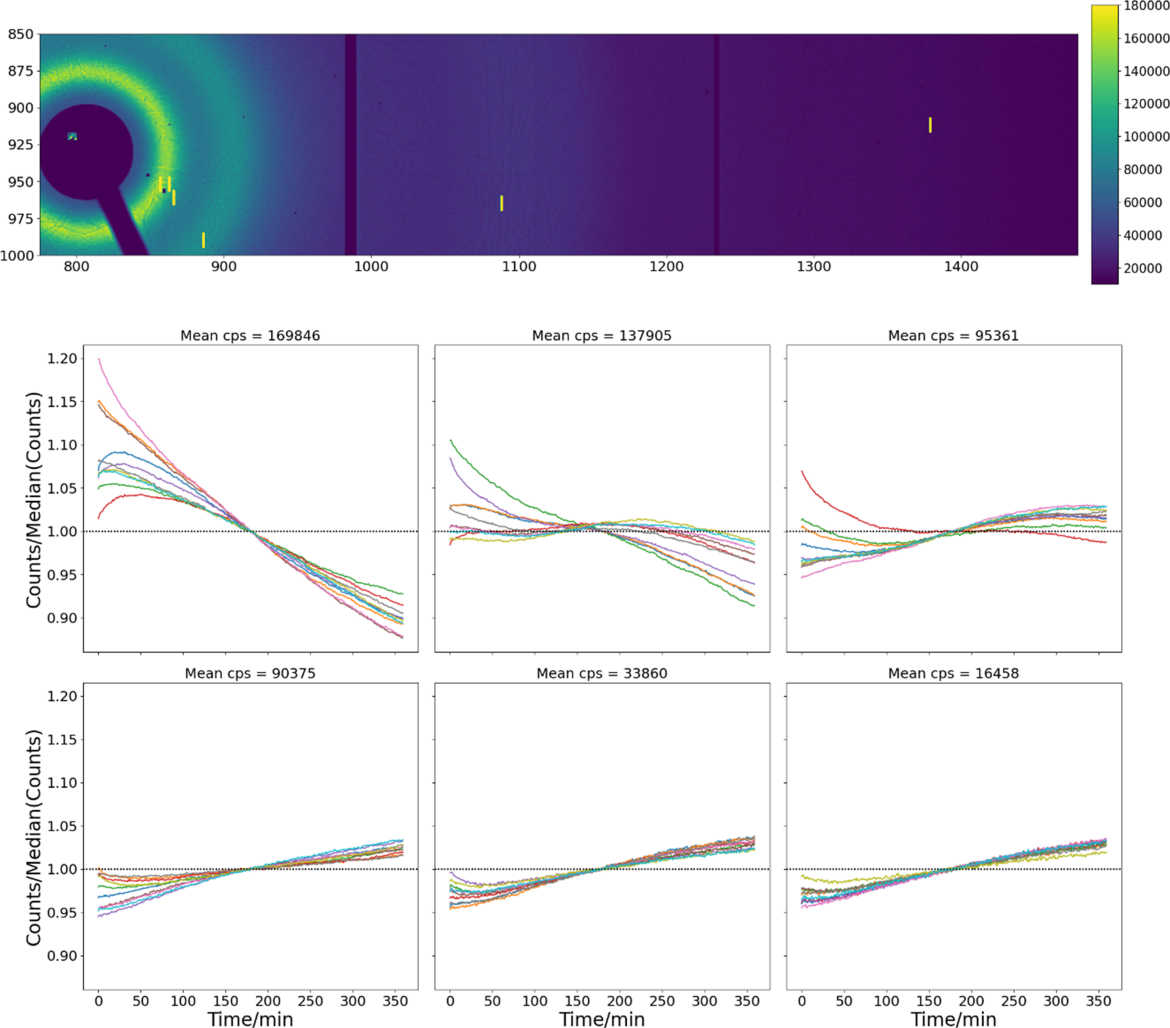
(Bottom) Each panel shows the time evolution of the counts in groups of pixels with similar count rates but having different distances from microstructural features on the detector, over 6 h. Data were taken each second, but averaged over 60 s in the figure. The signal of each pixel is divided by its median value. (Top) The location of the pixels on the detector.

To quantify the evolution of the response, we show in Fig. 10 ▸ the response of groups of pixels receiving roughly equivalent flux over 6 h. The differences in the time evolution of the flux between the different intensity groupings, as well as within each group, are significant. Within each group, the long-term behavior (>1 h) shows the same tendency if not the same magnitude. At the highest count rates measured here (170 kcps) all pixels show a continuous decline in response with little indication of approaching equilibrium values. At rates around 100 kcps, the tendency reverses, and all pixels show a gradual rise in response.

Initial responses within each group also vary. In the group receiving the strongest flux, some pixels show an initial rise, followed by a decrease with the rate gradually lowering. Others show a very strong initial drop, with the slope also decreasing with time. This behavior can be seen until about 100 kcps, below which no pixels show an initial rise, but some show an initial decrease before beginning to increase. In general, both the magnitude and the variation decrease with decreasing flux.

The variations within each group are related to the microstructure of the CdTe. Shown in each of the six panels in Fig. 10 ▸ are the signals associated with different pixels receiving roughly equivalent flux but at different distances from the linear features which appear in the images. These features are much less than the pixel size, as they appear pixelated in all cases.

**Figure 11:**
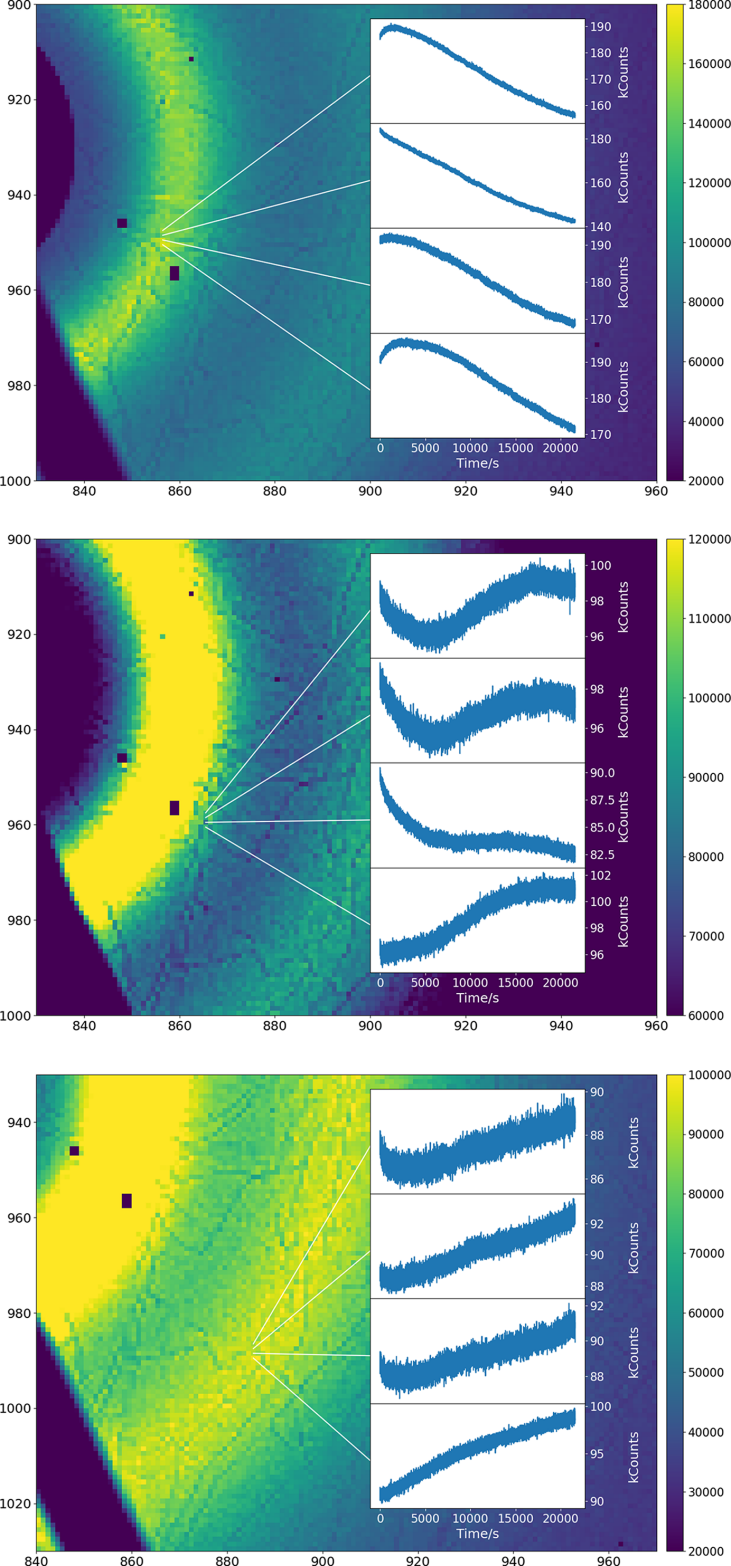
The time variation of different pixels receiving approximately equivalent counts. The images represent the data after 6 h of exposure, at the end of the displayed curves.

Fig. 11 ▸ (top) shows the response of 4 pixels in the strongly diffracting region. The second pixel, on the dark feature, shows a continuous and only slowly decreasing drop in response; the response after 6 h is about 75% of the initial value. The pixels away from this area all show an initial increase followed by a gradual decrease, with the effect strongest on the pixel not in contact with the grain boundary. After 6 h their response is 85–90% of the initial response (although the highest response is after about 2000 s).

Fig. 11 ▸ (middle) shows pixels receiving about 100 kcps. Again, the pixel on the dark feature shows the most extreme behavior, with the apparent count rate dropping rapidly before leveling out, but then dropping again. Pixels away from this area also show S-shaped curves, either dropping before rising and leveling out or else rising before flattening out. All pixels show low variation after about 18000 s. The variation of the pixels away from the dark feature is ∼5%, and that on it about 10%.

Fig. 11 ▸ (bottom) shows the data from the region with about 90 kcps. The differing initial behaviors are still present on a short time scale, and all pixels show a continuous increase thereafter, with the majority below 5% in variation. This behavior persists at lower count rates, with lower and lower magnitude.

The time-dependent effect is difficult to correct for analytically, due to the many parameters involved (energy, flux, history *etc.*). However it can be either (i) reduced to being a negligible effect, as values below ∼100 kcps produce drift on short (minutes) timescales well below 1%, particularly after the initial exposure, or (ii) characterized by periodic measurements of a standard material. The drifts are slow and monotonic and thus the pixel-by-pixel drifts can be calibrated against the unchanging signal of the standard. If the sample under study is a powder, it is sufficient to correct by the integrated powder pattern, thus rendering the correction less time consuming.

The PILATUS detector applies a rescaling scheme in order to extend the dynamic range beyond that where the response becomes nonlinear due to pile-up (Loeliger *et al.*, 2012 ▸). Count rates are thus rescaled on the basis of a lookup table (Kraft *et al.*, 2009 ▸; Trueb *et al.*, 2012 ▸; Trueb *et al.*, 2015 ▸). This method relies on the assumption that the flux on a given pixel is constant over the acquisition time. It has been pointed out that this is emphatically not valid in the case of single-crystal diffraction (Mueller *et al.*, 2012 ▸; Casanas *et al.*, 2016 ▸; Krause *et al.*, 2020 ▸). The variation here measured indicates that it is a good assumption in the case of scattering from a powder or amorphous sample.

### 3.5. Ghost signals

After extended exposure to high flux, the pixel response is modified, resulting in what appears as a ghost signal mirroring the strong scattering pattern. This effect slowly decays over a significant period of time (days), even if the bias voltage of the detector is repeatedly reset. At the current time, we are aware of no means to avoid the accumulation of this signal, beyond avoiding the collection of strong scattering data over long periods (depending on signal strength, this might mean seconds to days). Techniques to filter data at the time of azimuthal integration (Kieffer *et al.*, 2018 ▸) go some way to eliminate these ghost signals and alleviate the problem. Measurement and subtraction of backgrounds originating from these signals are problematic due to the nonlinear nature of their decay. This remains an outstanding issue to be treated, although such suggestions as translating the detector (Skinner *et al.*, 2012 ▸) are applicable in some cases, but only generally in the case of *ex situ* experiments. Best practice is thus to ensure that the measured count rate is sufficiently low so as to avoid the accumulation of these signals.

## 4. Examples

Here we mention a few examples of experiments carried out with the PILATUS 2M CdTe to demonstrate the quality of data that can be obtained with the detector and the corrections presented above.

### 4.1. Total scattering

The low noise and energy discrimination available are key aspects in the utility of the PILATUS for applications in total scattering [see Terban & Billinge (2022 ▸) for a recent review], where data quality relies notably on the accurate measurement of weak signals at high *q*. The detector characteristics of the PILATUS3 X CdTe detector, in particular the low noise, make it ideal for measuring the weak signals at high *q*, important for obtaining good results from total scattering data.

Two recently published studies demonstrate the quality of the pair distribution functions obtainable from very small, amorphous samples using the PILATUS detector. In the first case (Luo *et al.*, 2018 ▸; Luo *et al.*, 2021 ▸), 2 µm-thick films of metallic glass (MG) samples were measured in transmission; in the second case (Poonkottil *et al.*, 2022 ▸), 50 nm-thick films of Ru/RuO_2_ were measured in grazing-incidence geometry. In both cases, fine structural variations can be measured in extremely small, amorphous or nanocrystalline materials.

### 4.2. XRDCT

**Figure 12:**
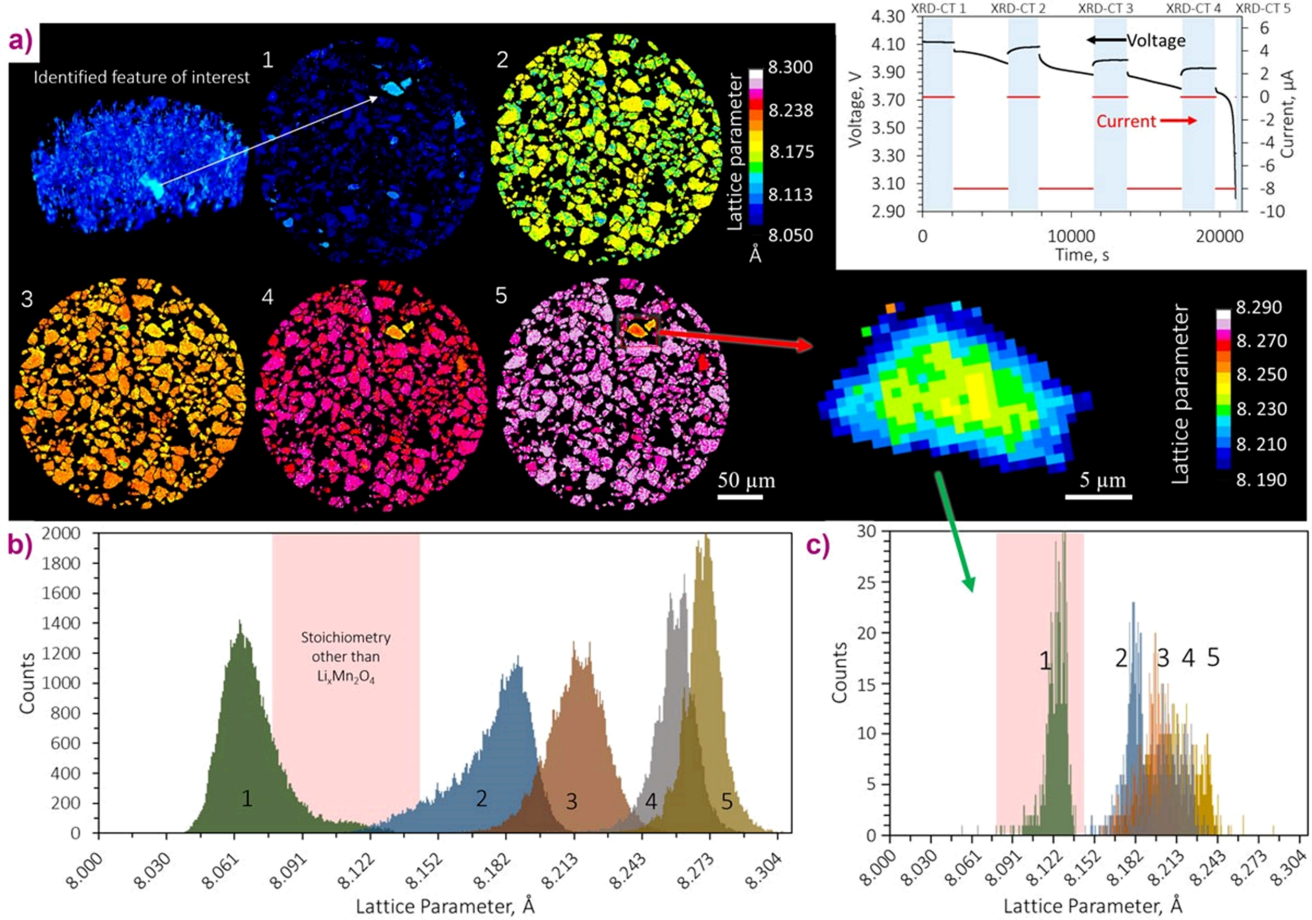
Sequential 1 µm-resolution XRDCT slices taken during discharge of the Li versus Li_*x*_Mn_2_O_4_ cell, showing the progression of li­thia­tion of the electrode phase. The discharge profile is shown as an inset and the blue regions indicate when XRDCT slices were recorded. A particle that appears to deviate from the bulk is isolated and magnified. (*b*) Histograms composed of the lattice parameter values assigned to each voxel in XRDCT slices 1–5. The pink region highlights the range of lattice parameter values over which a bi-phasic reaction of Li_*x*_Mn_2_O_4_ passes without occupying, *i.e.* a region that is not characteristic of the spinel Li_*x*_Mn_2_O_4_ stoichiometry. (*c*) Equivalent histograms for the single isolated particle showing that for XRDCT 1 the particle’s lattice parameter occupied a range that is not typical for Li_*x*_Mn_2_O_4_.

Prior to the adoption of this kind of detector, XRDCT [see *e.g.* Omori *et al.* (2023 ▸) for a recent review], a technique widely used at modern synchrotron radiation facilities, was limited to the study of static samples due to acquisition times of many hours. Here we present an example of *operando* high-speed XRDCT to probe crystallographic heterogeneities within a Li-ion electrode with a spatial resolution of 1 µm (Finegan *et al.*, 2020 ▸; Fig. 12 ▸). This capability to investigate dynamic processes with unprecedented spatial resolution has been possible thanks to the new high-speed high-efficiency photon-counting PILATUS3 X CdTe detector. XRDCT allowed amplification of signal from specific phases of interest by segmenting and distinctly quantifying phase fractions from regions where their presence was highest; using conventional point X-ray diffraction measurements, such detail would likely be lost in the noise.

## 5. Conclusion

In this paper we have demonstrated the excellent data quality achievable using the PILATUS3 X detector for diffraction from polycrystalline and amorphous materials, as long as care is taken in the collection and reduction of the data. Due to their characteristics as described in this paper, hybrid pixel detectors are rapidly becoming ubiquitous for high-energy elastic scattering applications. The majority of quality-limiting factors have been adequately treated, with the exception of the elimination of ghost signals from long-term collection of very strong scattering data.

This issue aside, it appears that, with respect to linearity, background and dynamic range, the quality of powder diffraction data that can be acquired with this sort of detector is approaching that which can be collected with a point detector/analyzer crystal arrangement, with an enormous increase in statistical quality.

## Supplementary Material

Application of corrections through the pyFAI API and supplementary figures. DOI: 10.1107/S1600576724010033/te5142sup1.pdf

## Figures and Tables

**Table 1:** Characteristics of different detectors used for diffraction and total scattering experiments

Detector type	Dark current	Spatial distortion	Homogeneity	Dynamic range	Readout frequency
Phosphor-coupled CCD	Low	High	Low–high	Low–moderate	High
Flat-panel	Very high	Very low	High	Moderate	High
Hybrid photon-counting (pixel)	None in photon-counting mode	Low	Low	High	Very high

**Table 2:** Comparison of PILATUS3 X CdTe and PerkinElmer XRD 1621 CN3 ES specifications

	PILATUS3 X CdTe 2M	Perkin Elmer XRD 1621 CN3 ES
Active area (W × H) (mm)	253.7 × 288.8	409.6 × 409.6
Pixel array (W × H)	1475 × 1679	2048 × 2048
Pixel size (W × H) (µm)	172 × 172	200 × 200
Inter-module gap (H/V) (pixels)	7/17	0
Intra-module gap (H) (pixels)	1	0
Count rate (max.) (photon s^−1^ pixel^−1^)	10^7^	∞
Energy range (keV) (from manufacturer)	15–∼120	20–15000
Frame rate (max.) (Hz)	250	15
Region of interest readout	Yes	No
Readout time (ms)	0.95	67
Dynamic range (bits)	20	12.8
Sensor material	CdTe	CsI
Sensor thickness (µm)	1000	500
Point-spread function (FWHM) (pixels)	1	1.5
