# Supplementary material for: Characterization and calibration of DECTRIS PILATUS3 X CdTe 2M high-Z hybrid pixel detector for high-precision powder diffraction measurements
Source: J Appl Crystallogr. 2025 Feb 1;58(Pt 1):76–86. doi: 10.1107/S1600576724010033 (PMC11798511; doi:10.1107/S1600576724010033)
Supplement: Supplementary file 1 [file j-58-00076-sup1.pdf]

JOURNAL OF
APPLIED
CRYSTALLOGRAPHY

Volume 58 (2025)

Supporting information for article:

Characterization and calibration of DECTRIS PILATUS3 X CdTe 2M high-*Z* hybrid pixel detector for high-precision powder diffraction measurements

Gavin B. M. Vaughan, Stefano Checchia and Marco Di Michiel

The corrections described in this paper are applied through the pyFAI API at different stages of azimuthal integration (www.silx.org/doc/pyFAI/latest/, 2024). This scheme was proposed by (Skinner et al., 2012), among others.

1) flat-field is applied to the raw image together with other factors (sensor absorption, solid angle, beam polarisation) contributing to the overall image normalisation

2) detector pixels are mapped onto radial and azimuthal arrays during the preparation of azimuthal integration. The mapping depends on the geometry of the particular experiment (wavelength, sample-detector distance, tilts) and on the properties of the detector itself. Spatial distortion is obtained by converting regularly gridded pixels in the idealised detector definition into calibrated pixel positions, typically stored in an hdf5 file.

3) after polar transformation of the normalised, spatially-corrected image, weighted averages of the pixels contributing to each radial bin become the final integrated intensities. At this stage, masked pixels are ignored during averaging based on the mask .

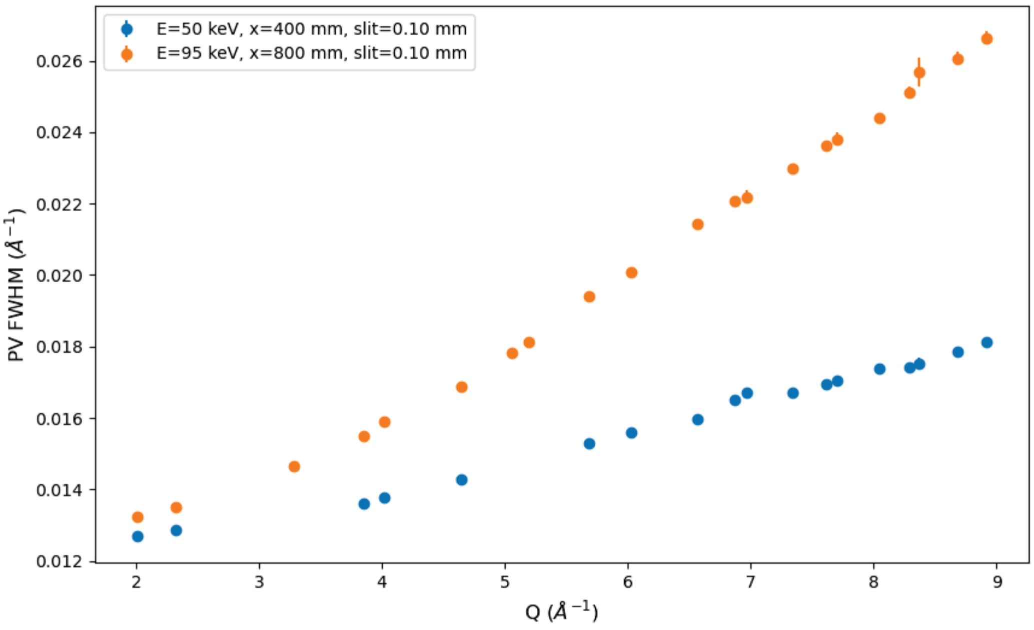

Figure S1 CeO_2 peak widths measured over the same Q-range for two different energies; the peak broadening is principally due to the monochromator band pass, as described in the paper.

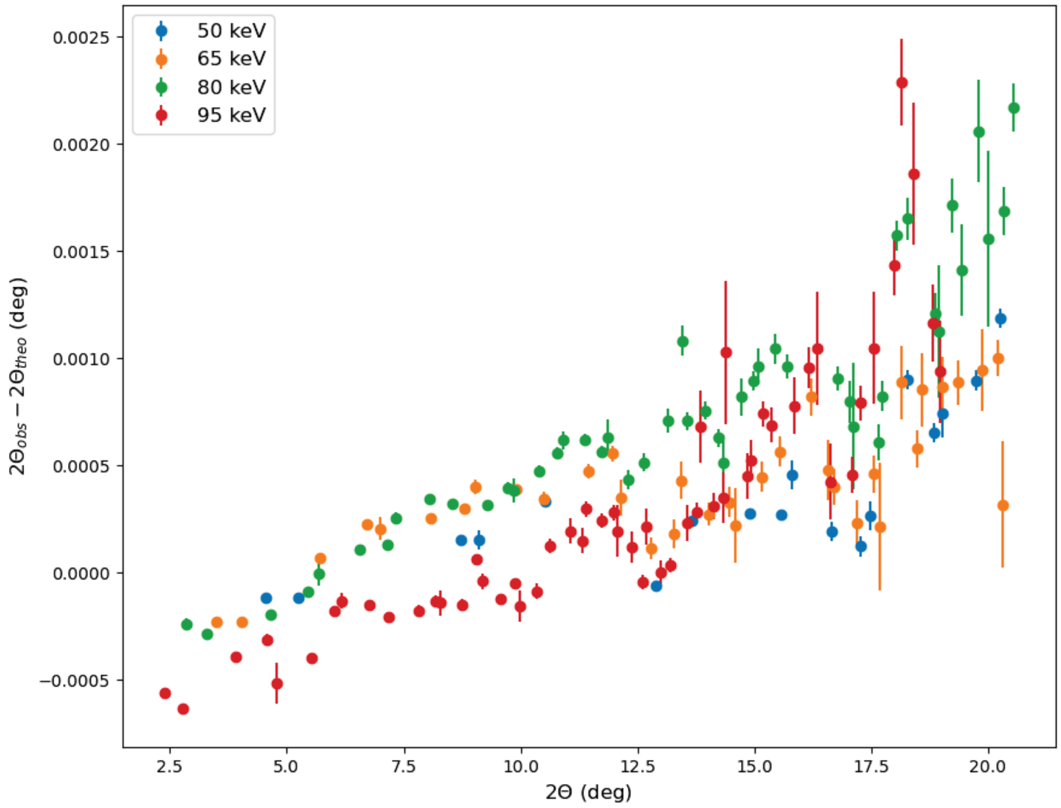

Figure S2 Peak offsets measured with respect to ideal cubic locations for CeO_2 at 4 energies

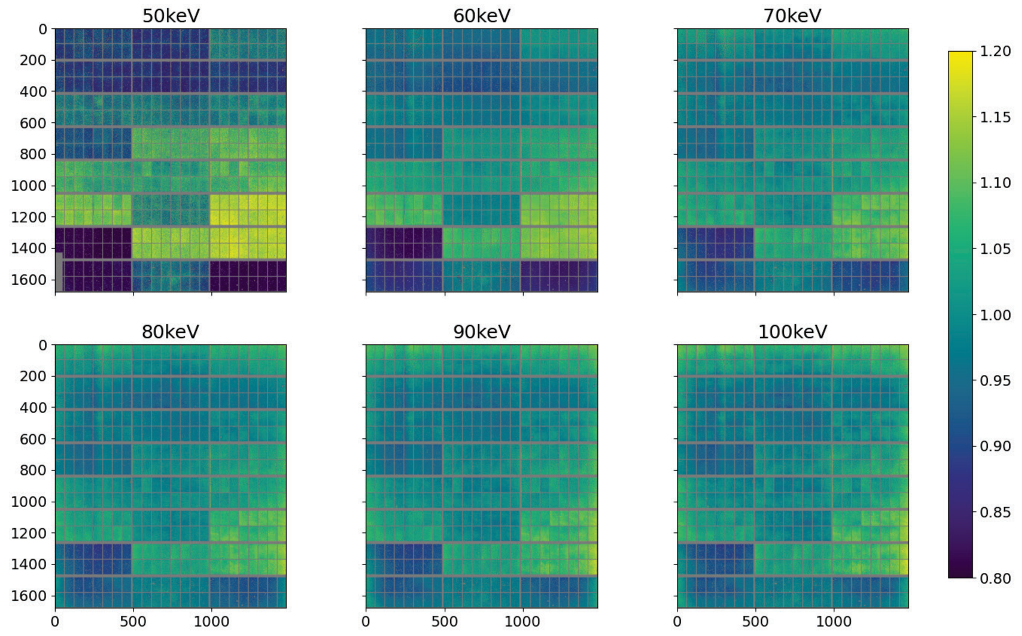

Figure S3 The flood field corrections measured between 50 and 100 keV

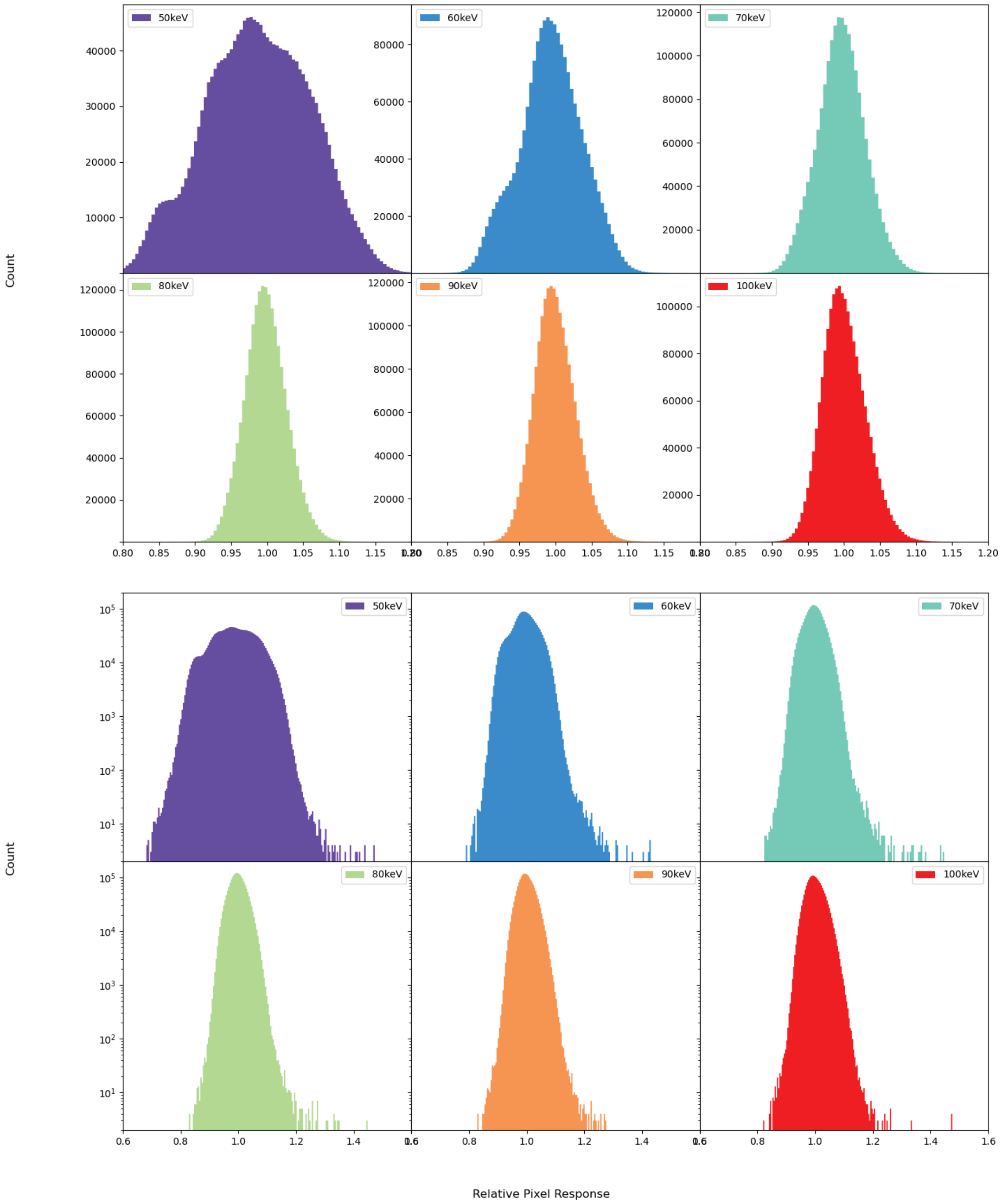

Figure S4 Distribution of 2M pixel responses as a function of energy, on linear (above) and log (below) scales. A value of 1.0 represents the mean detector response.

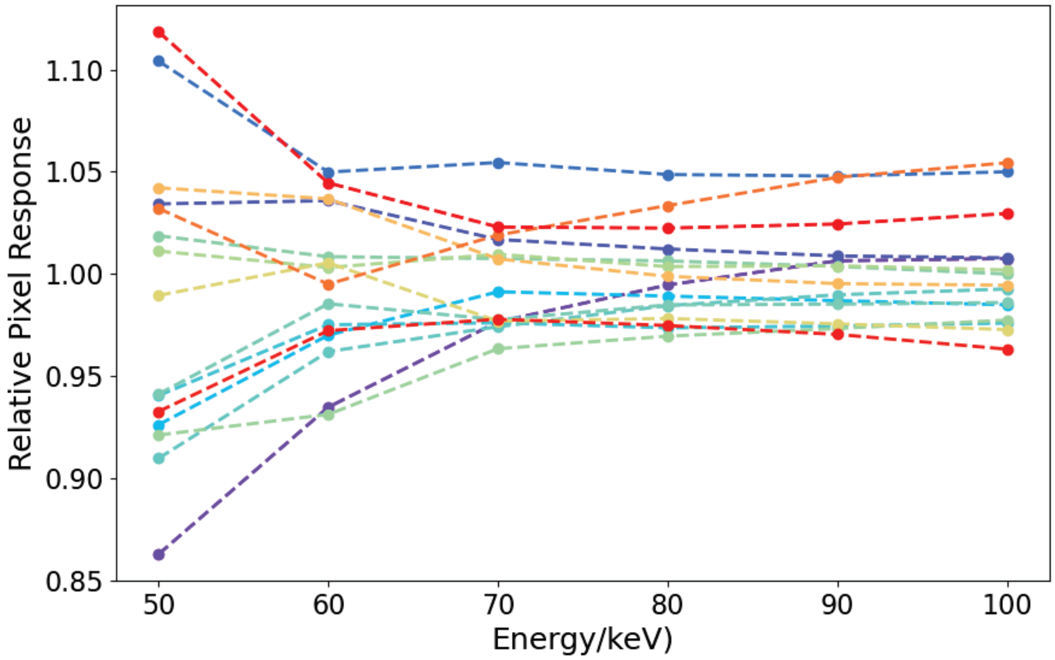

Figure S5 Variation of pixel responses with energy, for several randomly chosen pixels, displaying the largly monotonic but non-systematic variation.

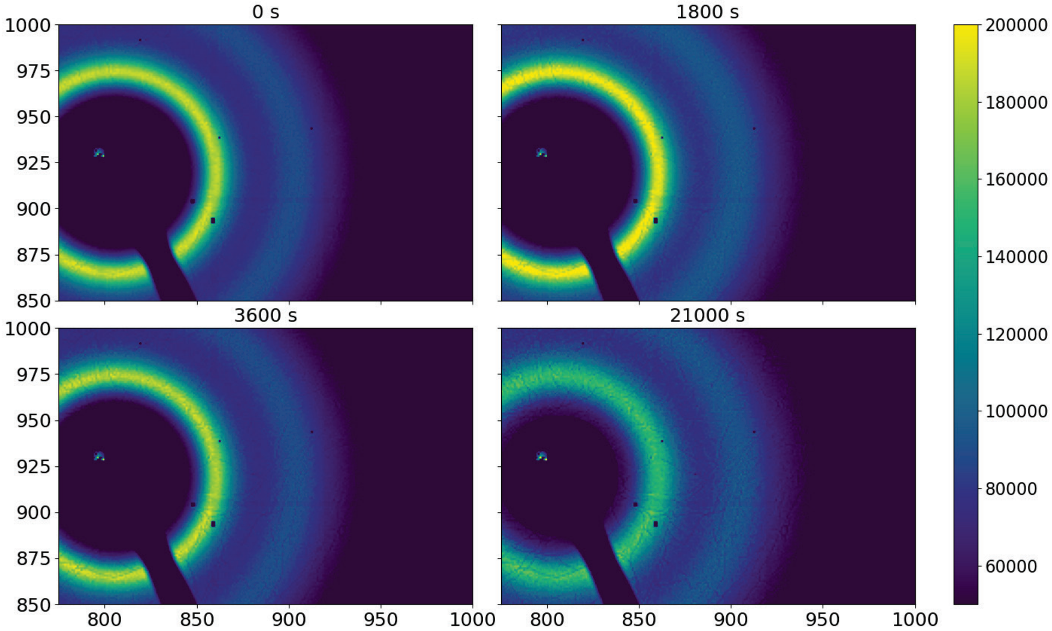

Figure S6 Appearance of distinct microstructure after prolonged exposure.
